# Multispectral optoacoustic tomography for *in vivo* detection of lymph node metastases in oral cancer patients using an EGFR-targeted contrast agent and intrinsic tissue contrast: A proof-of-concept study

**DOI:** 10.1016/j.pacs.2022.100362

**Published:** 2022-04-29

**Authors:** J. Vonk, J. Kukačka, P.J. Steinkamp, J.G. de Wit, F.J. Voskuil, W.T.R. Hooghiemstra, M. Bader, D. Jüstel, V. Ntziachristos, G.M. van Dam, M.J.H. Witjes

**Affiliations:** a Department of Oral & Maxillofacial Surgery, University of Groningen, University Medical Center Groningen, the Netherlands; b Chair of Biological Imaging at the Central Institute for Translational Cancer Research (TranslaTUM), School of Medicine, Technical University of Munich, Germany; c Institute of Biological and Medical Imaging, Helmholtz Zentrum München, Neuherberg, Germany; d Department of Surgery, University of Groningen, University Medical Center Groningen, the Netherlands; e Department of Pathology and Medical Biology, University of Groningen, University Medical Center Groningen, the Netherlands; f Department of Clinical Pharmacy and Pharmacology, University Medical Center Groningen, University of Groningen, Groningen, the Netherlands; g Institute of Computational Biology, Helmholtz Zentrum München, Neuherberg, Germany; h Department of Nuclear Medicine and Molecular Imaging, University Medical Center Groningen, University of Groningen, Groningen, the Netherlands; i AxelaRx / TRACER B.V., Groningen, the Netherlands

## Abstract

Oral cancer patients undergo diagnostic surgeries to detect occult lymph node metastases missed by preoperative structural imaging techniques. Reducing these invasive procedures that are associated with considerable morbidity, requires better preoperative detection. Multispectral optoacoustic tomography (MSOT) is a rapidly evolving imaging technique that may improve preoperative detection of (early-stage) lymph node metastases, enabling the identification of molecular changes that often precede structural changes in tumorigenesis. Here, we characterize the optoacoustic properties of cetuximab-800CW, a tumor-specific fluorescent tracer showing several photophysical properties that benefit optoacoustic signal generation. In this first clinical proof-of-concept study, we explore its use as optoacoustic to differentiate between malignant and benign lymph nodes. We characterize the appearance of malignant lymph nodes and show differences in the distribution of intrinsic chromophores compared to benign lymph nodes. In addition, we suggest several approaches to improve the efficiency of follow-up studies.

## 1 Introduction

In oral cancer patients, the presence of lymph node (LN) metastases is one of the most important predictors of poor prognosis, with a reported decrease of survival by as much as 50% [1], [2]. Clinical and radiological staging (*i.e*., magnetic resonance imaging (MRI) or computed tomography (CT)) of the neck is performed to determine the most appropriate surgical strategy. However, in 20–30% of the patients with a clinically negative neck (cN0), occult LN (micro-)metastases are present at the time of surgery, demonstrating that current preoperative imaging methods lack the necessary sensitivity for detection of LN metastasis. When the probability of occult metastasis is > 20%, an elective neck dissection or sentinel node biopsy is warranted for a better oncological outcome [3], [4], [5]. Yet, many of these patients do not have metastatic LNs on definitive histopathological analysis (*i.e*., 70–80% of early-stage oral cancer patients with cN0), which implies significant overtreatment that is associated with morbidity, such as pain, shoulder dysfunction, and impaired quality of life [6], [7], [8].

The drive towards a patient-specific and less invasive surgery in oral cancer patients requires improved preoperative detection of LN metastases. Current imaging methods mainly rely on the LN size, the presence of central necrosis, the appearance of extranodal extension and the contrast enhancement on CT or MRI [9]. Often, small LNs are not detected, while these are the ones that harbor occult metastases. Since biochemical changes occur before morphological changes in disease, molecular imaging approaches may have a diagnostic advantage and higher sensitivity to detect LN metastases in an early stage.

Multispectral optoacoustic tomography (MSOT) is an emerging non-invasive imaging method exploiting the optoacoustic effect, where ultrasound waves are produced in tissue in response to the absorption of short laser pulses [10]. MSOT enables visualization of both intrinsic tissue chromophores and administered contrast agents at imaging depths of several centimeters in tissue [11], [12]. The potential of MSOT has been shown for multiple clinical indications, such as sentinel lymph node mapping [13] and assessment of inflammatory bowel disease [14], Duchenne muscular dystrophy [15], cardiovascular disease [16], [17], [18], [19], thyroid disease [20], [21] and breast cancer [22], [23], [24]. Specifically, oxy- (HbO_2_) and deoxy-hemoglobin (HbR) absorption properties allow visualization of tumor-related angiogenesis and hypoxia [25], [26], [27], [28], [29]. Preclinical studies suggest that the presence of angiogenesis, the distortion of peripheral LN vasculature, and the decreased oxygen saturation resulting from tumor hypoxia can be possible indicators for LN malignancy [30], [31]. To improve the specificity of MSOT, near-infrared tumor-specific fluorescent tracers are considered as they exhibit a reasonable molar extinction coefficient and low fluorescence quantum yield, benefitting the optoacoustic signal generation [32], [33]. For example, peritumoral administration of indocyanine green allowed for the identification of sentinel LNs with MSOT, however, non-invasive evaluation of the LN metastatic status remains an open problem [13], [34]. MSOT could potentially detect malignant LNs when combined with tumor-specific contrast agents. Multiple studies have shown the use of Epidermal Growth Factor Receptor (EGFR)-targeted fluorescent tracers (*e.g.*, panitumumab-800CW and cetuximab-800CW) to detect malignant LNs *ex vivo* in oral cancer patients using fluorescence molecular imaging [35], [36]. A recent study by Nishio et al. demonstrated the capability of MSOT to visualize panitumumab-800CW and thus differentiate benign from malignant LNs in an *ex vivo* setting [37].

Here, we are the first to demonstrate the use of MSOT for *in vivo* imaging of neck LNs in oral cancer patients. We characterize the optoacoustic properties of the tumor-specific fluorescent tracer cetuximab-800CW in a tissue-mimicking phantom and determine an estimated minimal detectable concentration *in vitro*. Next, we image the LNs *in vivo* before and after cetuximab-800CW administration in patients and identify limitations that prevent a successful detection of the tracer. Furthermore, we analyze the features of the imaged LNs provided by resolving the intrinsic chromophores, HbO_2_ and HbR. Although the small sample size prevents a large-scale evaluation, we observe increased variance of HbR distribution in malignant LNs and demonstrate that clinically significant features of LNs can be observed with handheld MSOT, providing the rationale and need for a larger clinical trial to substantiate these findings.

## 2 Methods

### 2.1 Study design

This single center, proof-of-concept study was performed at the University Medical Center Groningen, Groningen, the Netherlands. Approval was obtained at the Institutional Review Board of the hospital and the Central Committee on Research Involving Human Subjects. All patients provided written informed consent prior to any study-related procedure. The study was performed in compliance with the Dutch Act on Medical Research involving Medical Subjects and the Declaration of Helsinki (adapted version 2013, Fortaleza, Brazil). The trial was registered at clinicaltrials.gov (NCT03757507).

### 2.2 Participants

Patients ≥ 18 years eligible for inclusion in this study had histology-confirmed oral cancer and were already included in the clinical trial of our group on fluorescence-guided surgery for margin assessment (ICON-study, NCT03134846). Exclusion criteria are described earlier and are provided in supplemental materials [38]. Two days prior to surgery, patients were administered with 15 mg cetuximab-800CW preceded by 75 mg unlabeled cetuximab to prevent rapid plasma clearance and to occupy off-target receptors, previously determined as the optimal dosing strategy for primary tumor imaging [38]. Subjects that underwent a surgical procedure of the neck with concurrent primary tumor surgery were included in this study, as histopathology was the reference standard of *in vivo* MSOT. Clinical and pathological TNM staging was performed according to the 8th edition of the American Joint Committee on Cancer (AJCC) criteria.

### 2.3 Production and characterization of cetuximab-800CW

Cetuximab-800CW was manufactured in the University Medical Center Groningen according to good manufacturing-practice guidelines, as previously described [39]. Briefly, cetuximab (Erbitux®) and IRDye800CW NHS Ester (LI-COR Biosciences, Lincoln, NE, USA) were conjugated under regulated conditions with a dye/antibody ratio of 1:2. Cetuximab-800CW was formulated in a sodium phosphate solution at a concentration of 1 mg/mL.

Prior to the clinical study, the optoacoustic spectra of cetuximab-800CW and IRDye800CW were determined with a hybrid ultrasound-MSOT system (see subsection 2.4). A tissue-mimicking phantom was fabricated using 300 mL deionized water, 2% agarose and 6% intralipid to mimic the optical properties of biological tissue. At 1 cm depth, polyethylene tubes with a diameter of 3 mm were inserted. These were filled with cetuximab-800CW 1 mg/mL and IRDye800CW with an optical density of 2 as a reference. The phantom was placed in a water bath to ensure optimal coupling between the transducer and the phantom and imaging was performed at 660–900 nm with 5 nm step size. The tubes were manually segmented in the reconstructed optoacoustic images and the spectra of cetuximab-800CW and IRDye800CW were determined as the mean absorption in the segmented regions. Supplemental Fig. S1 shows an overview of the experiment and example US and MSOT images.

To verify the linear relationship between the cetuximab-800CW concentration and the optoacoustic signal strength, and estimate the minimal detectable concentration, a two-fold dilution series down to 1.6 μM was imaged in the phantom. Six wavelengths were used here (700, 730, 760, 780, 800 and 850 nm), to match the *in vivo* imaging procedure. Non-negative least squares linear spectral unmixing using spectra of HbR, HbO_2_, and cetuximab-800CW was applied to compute the cetuximab-800CW concentrations in the optoacoustic images (see subsection 2.5). Again, the mean of the unmixing coefficients in the region of interest (ROI) was computed to estimate the concentration measured by MSOT.

### 2.4 In vivo imaging protocol

All imaging procedures were performed with a hybrid ultrasound-MSOT system (MSOT Acuity Echo prototype; iThera Medical GmbH, Munich, Germany). This system comprises a fast-tunable Nd:YAG laser (25 Hz pulse repetition rate, 4–7 ns pulse duration) and a 256-element 125° arc-shaped ultrasound transducer array (3.4 MHz central frequency). The maximum output energy of this system is in accordance with the American National Standards Institute safety limit for laser exposure [40].

All patients were imaged in a supine position with the neck in hyperextension and turned away from the imaging area. The neck was explored for LNs using the inbuilt ultrasound guidance of the system. Of all identified LNs, a video was obtained of ~10 s. The anatomical location of each identified LN within the various levels of the neck was mapped. MSOT images were acquired at six wavelengths (700, 730, 760, 780, 800 and 850 nm) selected to reflect the characteristics of the absorption spectra of HbR, HbO2, and IRDye800CW. Imaging was performed in a dedicated MSOT-imaging room following all safety regulations for safe use of class IV lasers (*e.g.*, laser interlock system, safety goggles). Patients were asked for any symptoms present during or after imaging, and their skin was visually inspected.

### 2.5 In vivo imaging analysis

**Fig. 1:**
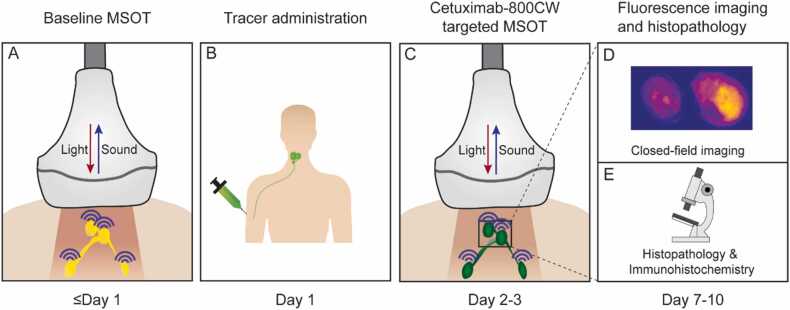
**Study workflow.** After baseline multispectral optoacoustic tomography (MSOT), all patients were intravenously administered with 75 mg cetuximab followed by 15 mg cetuximab-800CW. Two days later, MSOT was performed again. After surgical removal of the lymph nodes, single lymph nodes were imaged during pathology processing of the nodal specimen and correlated with final histopathology. Abbreviations: MSOT, multispectral optoacoustic tomography.

The MSOT images were reconstructed from the filtered acoustic signals (Butterworth filter, 0.5–12 MHz) with a model-based approach using a LSQR algorithm with a non-negativity constraint and Shearlet L^1^-regularization [41], [42], [43]. The regularization parameter was tuned to be 0.01 using an L-curve. The model accounted for the acoustic and electrical properties of the probe summarized as total impulse response correction [44], [45]. To improve the signal-to-noise ratio, three consecutive MSOT frames were reconstructed and averaged [24]. The LNs were manually segmented on the ultrasound images generated by the scanner along with MSOT to specify ROIs for further analysis. Fig. 1.

**Fig. 2:**
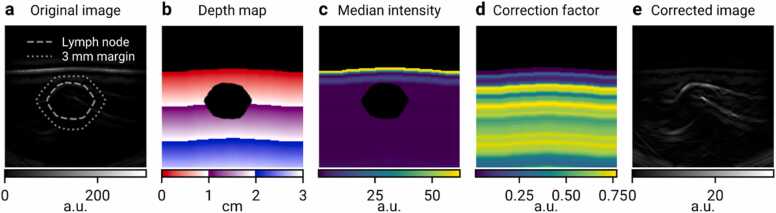
**Depth correction procedure** demonstrated on the image of malignant lymph node (LN) M1. a) Uncorrected optoacoustic image (λ = 700 nm) demonstrates the signal intensity decaying with depth. Only the top layer of the skin is visible, all other structures have too low intensity to be seen. The location of the LN and its 3 mm margin are denoted by dashed and dotted lines, respectively. b) Depth map. Pixels above the skin surface and inside the LN region are excluded. c) Map of median intensities. Each pixel shows the median intensity over all pixels in the image that are equally deep. Pixels in the LN region are excluded from the computation. As in (a), intensity decaying quickly with depth can be seen. d) Map of reciprocals to the median intensities shown in (c), which are used as multiplicative correction factors. e) Depth-corrected image, obtained by multiplying images (a) and (d), shows that the approach balances the signal intensities over the whole field of view.

A depth-gain correction procedure (Fig. 2) was applied to avoid depth-effects biasing our quantitative analysis. Given an original MSOT image (Fig. 2a) $I_{\mathrm{orig}}:\Omega \subset R^{2}\times \Lambda \to R$, where Ω denotes the set of image pixels and Λ the set of acquired wavelengths, a depth correction factor c is computed, as a function of depth D (Fig. 2b) and wavelength λ, to be the median (Fig. 2c) of all pixels in the same depth which are not inside the LN ROI $\Omega_{\mathrm{LN}}$:

$$c\left(D,\;\lambda \right)=\mathrm{median}\left(I_{\mathrm{orig}}\left(x,\;\lambda \right)|x\in \Omega \setminus \Omega_{\mathrm{LN}}\wedge d\left(x\right)=D\right).$$

Here, $d\left(x\right)$ represents the depth of a pixel x. Then, the corrected image $I_{\mathrm{corr}}$ (Fig. 2e) is defined as

$$I_{\mathrm{corr}}\left(x,\lambda \right)=\frac{I_{\mathrm{orig}}\left(x,\;\lambda \right)}{c\left(d\left(x\right),\;\lambda \right)+\varepsilon }\;,$$

where ε is a correction term to ensure numerical stability in the regions of the image with near-zero values. In our analysis, ε was set to 1. Map of correction factors ${\left(c+\varepsilon \right)}^{-1}$ is shown in Fig. 2d. Furthermore, to limit the artifacts of discretization of the depth map, the function $c\left(\cdot ,\lambda \right)$ was smoothened with a Gaussian kernel with σ = 0.3 mm.

The concentrations of chromophores (HbR, HbO_2_, lipids, and cetuximab-800CW) were estimated using non-negative linear spectral unmixing. Specifically, we obtained the vector of concentrations a for each pixel x with spectrum $p\equiv {\left[I_{\mathrm{corr}}\left(x,\lambda_{1}\right),\ldots ,I_{\mathrm{corr}}\left(x,\lambda_{\left|\Lambda \right|}\right)\right]}^{T}$ by solving the non-negative least squares problem $\underset{a\geq 0}{\mathrm{argmin}}{\left\|\mathrm{Sa}-p\right\|}_{2}$, where S is a matrix with the absorption spectra of the four chromophores as columns [46].

The distributions of HbR and HbO_2_ concentrations in each LN were characterized using their variance relative to 3 mm margins around the LNs (Fig. 2a) as $\mathrm{Var}\left(H{\mathrm{bR}}_{\mathrm{LN}}\right)/\mathrm{Var}(H{\mathrm{bR}}_{\mathrm{Margin}})$, where ${\mathrm{HbR}}_{\mathrm{LN}}$ and $\mathrm{Hb}R_{\mathrm{Margin}}$ are the sets of HbR unmixing coefficients for all pixels in the respective ROIs, and a corresponding formula is applied to HbO_2_ as well. This approach has twofold benefit for the robustness of our analysis. First, the use of relative variance eliminates any subject-dependent linear effects (*i.e*., constant offset and multiplicative bias) on the results that might be caused *e.g.*, by varying melanin content between the subjects [47]. Second, normalizing by variance of neighborhood, whose size is proportional to the size of the LN, minimizes the possibility that an increased value is observed solely due to increased size of a LN.

### 2.6 *Ex vivo* analysis of surgical specimen

Directly after the surgical excision, the nodal specimen was fixed in formalin at the Department of Pathology for at least 24 h. According to the standard of care, the nodal specimen was divided in anatomic neck LN levels, and for each level single LNs were identified by visual and tactile inspection. The location of each identified LN was annotated on a white-light image of the complete nodal specimen to allow for correlation between *in vivo* imaging and pathology. LNs were bisected when large enough and subsequently collected in cassettes. All LNs were imaged in a closed-field fluorescence imaging system (Pearl Trilogy®, LI-COR BioSciences) to ensure control of imaging parameters. The protocol for fluorescence imaging of LNs has been described previously [36], [48].

Fluorescence images of the formalin-fixed LNs were used to draw ROIs to delineate the LNs and calculate the mean fluorescence intensity, defined as total counts per ROI pixel area (signal/pixel). Subsequently, LNs were embedded in paraffin and 4 µm sections were cut for hematoxylin and eosin (H&E) staining. According to the standard of care, a head and neck pathologist microscopically examined all tissue sections to determine the number of resected LNs and their metastatic state.

### 2.7 Statistical analysis

Statistical analyses were conducted using Graphpad Prism version 8. Due to the limited sample size of this study, all data was considered non-normally distributed. The Mann-Whitney *U* test was used to analyze distributions of HbO_2_ and HbR. A p-value < 0.05 was considered statistically significant.

## 3 Results

### 3.1 Optoacoustic characterization of cetuximab-800CW in a phantom

**Fig. 3:**
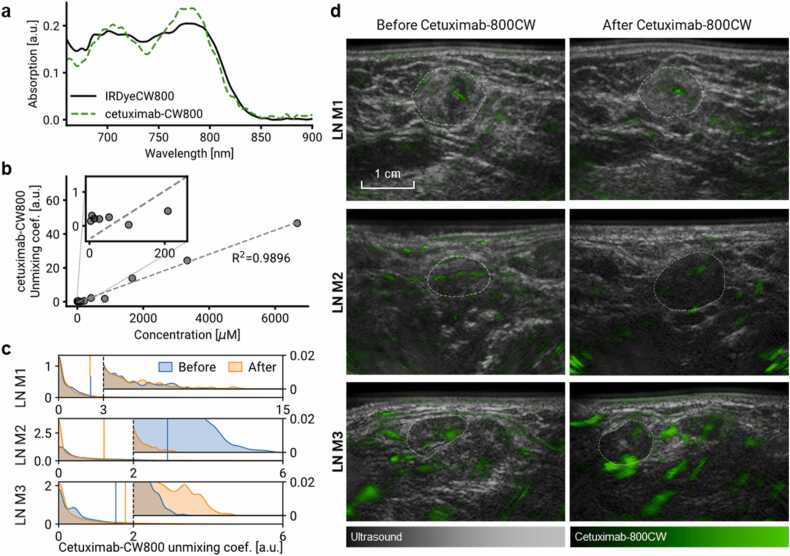
**Optoacoustic characterization of cetuximab-800CW and its detection in malignant lymph nodes*****in vivo*****.** a) Absorption spectra of IRDye800CW and cetuximab-800CW solutions in a tissue-mimicking phantom, acquired with the MSOT Acuity Echo. b) Unmixing coefficient of cetuximab-800CW in optoacoustic phantom images as a function of its concentration, showing a linear relationship. c) Distribution of unmixing coefficients of cetuximab-800CW in three malignant lymph nodes *in vivo* before and after injection of the contrast agent. Insets show the right tails of the distributions where a potential localized increase could be observed. Vertical lines denote 95th percentiles. d) Hybrid visualizations of cetuximab-800CW unmixing coefficients (green) overlapping greyscale ultrasound, providing both morphological and molecular information on the tissue of interest. Cetuximab-800CW signal cannot reliably be detected with the current setup since it is not specifically present in the malignant LNs. The signal visualized throughout the image is most likely due to errors of linear unmixing, and presumably originates from hemoglobin contrast. In addition, administration of cetuximab-800CW does not result in an apparent increase in optoacoustic signal within the malignant LNs. LN locations are denoted by dashed lines. Abbreviations: LN M, malignant LN.

First, we characterized the spectrum of cetuximab-800CW in MSOT and we verified a linear relationship between the contrast agent concentration and the unmixing coefficients computed from MSOT images. Fig. 3a displays the spectra of cetuximab-800CW and unconjugated IRDye800CW as recorded by MSOT *in vitro* using a tissue-mimicking phantom. The spectrum of cetuximab-800CW has peaks at 780 nm and 700 nm, the former mirroring a peak of IRDye800CW and the latter related to the forming of H-aggregates [37], [49], [50]. Fig. 3b shows that the MSOT unmixing coefficients of cetuximab-800CW increase linearly with its concentration in the phantom (R^2^ =0.9896) and can be reliably distinguished from the background signals at concentrations above 400 μM. At concentrations below 400 μM, the linear relationship does not hold (R^2^ <0).

### 3.2 Participants

Seven patients participated in this clinical study. Table 1 summarizes patient demographics and clinical characteristics. All patients received the study drugs and completed the imaging protocol. No adverse events or complaints were reported related to the MSOT procedure. Four patients presented with a clinically negative neck, and three patients with a clinically positive neck. Four patients underwent an elective neck dissection, of which one was extended to a modified radical neck dissection as a malignant LN was intraoperatively identified through frozen section biopsy. Three patients underwent a modified radical neck dissection, of which one also received an elective neck dissection on the contralateral side. The study was ended prematurely as the primary endpoint could be assessed earlier.

**Table 1:** Patient characteristics.

**Patient**	**Sex**	**Ethnicity**	**Age**	**Tumor location**	**cTN**	**pTN**	**No. of metastases**
**1**	Female	White	65	Tongue	cT2N0	pT2N1	1
**2**	Female	White	78	Mandible	cT4N0	pT4N0	NA
**3**	Female	White	63	Maxilla	cT3/4N0	pT2N0	NA
**4**	Female	White	66	Mandible	cT3–4aN1	pT4aN2a	1
**5**	Male	White	29	Tongue	cT1N0	pT1N0	NA
**6**	Female	White	65	Tongue	cT4aN2b	pT4aN3b	7
**7**	Female	White	47	Mandible	cT4aN0	pT4aN0	NA

Summary of patient, imaging and LN characteristics. Abbreviations: cTN, clinical tumor and nodal stage; pTN, pathological tumor and nodal stage; NA, not applicable.

### 3.3 *In vivo* MSOT of lymph nodes in patients with oral cancer

Due to the nature of the surgical procedure, exact tracking of all imaged LNs between *in vivo* imaging and histopathology was not possible. Only a subset of LNs where the metastatic status could be assigned with certainty was included in the *in vivo* image analysis. Specifically, we included only LNs of patients showing no malignancies at all at the histopathology, classified as benign (n = 11), and malignant LNs that were either palpable or close to anatomical landmarks and thus could be tracked until histopathology (n = 3). Five benign LNs that allowed correlation with histopathology were excluded from the analysis due to image quality issues: in one case the LN was obscured by skin reflection artifacts and four cases had surface contact issues causing strong artifacts while also having the view on the LN obscured by the sternocleidomastoid muscle. One malignant case (M1) was also partially affected by the skin reflection artifact but could still be used for the analysis after excluding the affected pixels. Supplemental Fig. S2 demonstrates these problems in detail. Supplemental Fig. S3 summarizes the data exclusions. An overview of the LNs analyzed with MSOT is provided in Table 2.

**Table 2:** Overview of lymph nodes analyzed with MSOT.

**Lymph node**	**Patient**	**Status**	**Depth (mm)**	**Diameter (US; mm)**	**Diameter (histology; mm)**
**M1**	4	Malignant	4.1	12.9	13.8 × 8.4
**M2**	6	Malignant	3.7	11.3	10.4 × 9.5
**M3**	6	Malignant	2.9	8.1	11.4 × 9.3
**B1**	1^a^	Benign	12.0	18.7	N/A
**B2**	1^a^	Benign	5.7	8.3	N/A
**B3**	7	Benign	10.4	7.7	N/A
**B4**	7	Benign	5.0	6.0	N/A
**B5**	1^a^	Benign	11.6	20.2	N/A
**B6**	2	Benign	7.1	16.6	N/A

a this patient presented with one solitary metastasis in the contralateral side of the neck.

Analyzing the *in vivo* images, we observed that cetuximab-800CW could not be reliably detected in our setup. Fig. 3c shows the distributions of cetuximab-800CW unmixing coefficients in three malignant LNs pre- and post-injection. Comparing the tails of the cetuximab-800CW unmixing coefficient distributions, where a localized increase of coefficients would be apparent, we observed that the LN M3 exhibited an increase, LN M2 showed a decrease and LN M1 remained unchanged. We obtained the same result also when comparing the 95th percentiles or considering the means of the upper top 10% of the coefficient values, as reported in the earlier *ex vivo* study [37]. Fig. 3d shows visualizations of the cetuximab-800CW signal as a green overlay on the grayscale ultrasound images. The post-injection scan of the LN M3 clearly shows patches of increased signal, albeit a discrepancy exists between imaging angles pre- and post-injection, preventing us from conclusively proving the possibility to detect cetuximab-800CW *in vivo*.

**Fig. 4:**
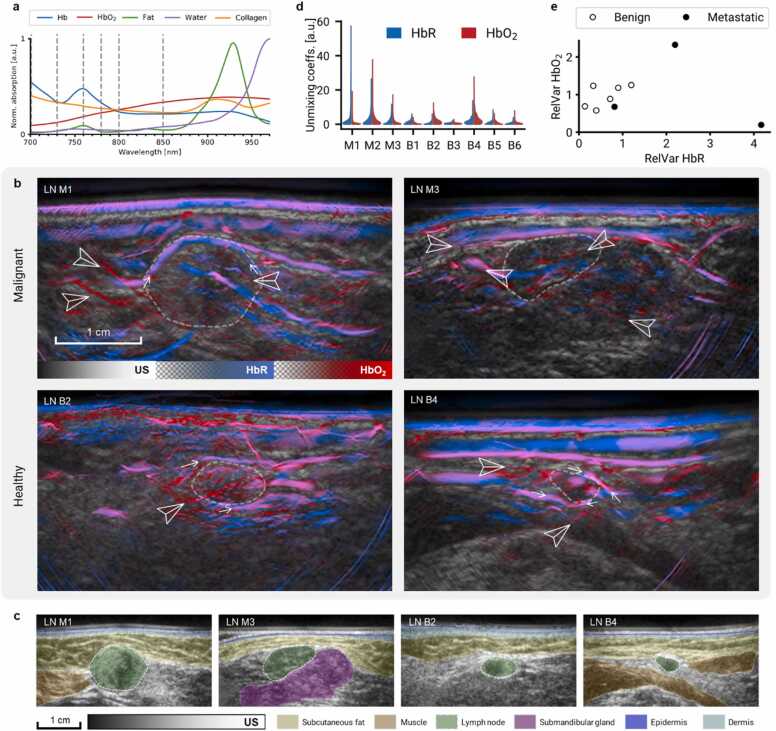
**Intrinsic contrast in lymph nodes.** a) Absorption spectra of main endogenous chromophores in the illumination range of the MSOT Acuity Echo scanner. Dashed lines denote the wavelengths used for the multispectral image acquisition (700, 730, 760, 780, 800, 850 nm). b) Ultrasound and linear spectral unmixing images of lymph nodes (LNs) M1, M3, B2, and B4. Arrowheads mark peripheral, feeding, and intranodal blood vessels. Small arrows mark signal enhancement around the LNs. Scalebar applies to all four images. Separate HbR and HbO_2_ maps are provided in Fig. S4. Strong HbR signal in the epidermis can be attributed to the presence of melanin. c) Ultrasound images of the LNs displayed in (b) with localization of surrounding anatomical landmarks. d) Distribution of Hb and HbO_2_ unmixing coefficients in three malignant LNs (M1–3) and six benign LNs (B1–6). e) Variance of chromophore concentrations in malignant and benign LNs relative to their 3 mm margins. Malignant LNs exhibit large increase of relative deoxy-hemoglobin variance. Abbreviations: LN M, malignant lymph node; LN B, benign LN; HbO2, oxyhemoglobin; HbR, deoxyhemoglobin.

On the other hand, using MSOT we could visualize intrinsic chromophores and related features in lymph nodes *in vivo*. Fig. 4a shows the absorption spectra of the main tissue chromophores in the wavelength range of MSOT. Dashed lines denote the wavelengths acquired during this study, allowing distinguishing HbR and HbO_2_. Fig. 4b shows images of malignant LNs (M1 and M3) and benign LNs (B1 and B4) with distinct features, such as vasculature (peripheral, feeding, and internodal; marked by arrowheads) and enhancement of HbR and HbO_2_ signals in the LN borders (small arrows), although we could not establish apparent differences between malignant and benign LNs. Separate HbR and HbO_2_ images are provided in Supplemental Fig. S4. Fig. 4c shows US images with localization of adjacent anatomical structures to provide a better spatial context to the displayed MSOT images.

Quantitatively, malignant LNs exhibited larger variance of HbR coefficients than the benign ones. The distributions of the unmixing coefficients of HbR and HbO_2_ throughout the LN ROIs are shown in Fig. 4c. Fig. 4d shows the variances of HbR and HbO_2_ distributions of malignant and benign LNs relative to their margins. Malignant LNs exhibit significantly higher variance of HbR than benign ones (p = 0.047, n = 9). No significant difference in HbO_2_-variance was observed between malignant and benign LNs (p = 0.349, n = 9).

### 3.4 *Ex vivo* analysis of surgical specimens

**Fig. 5:**
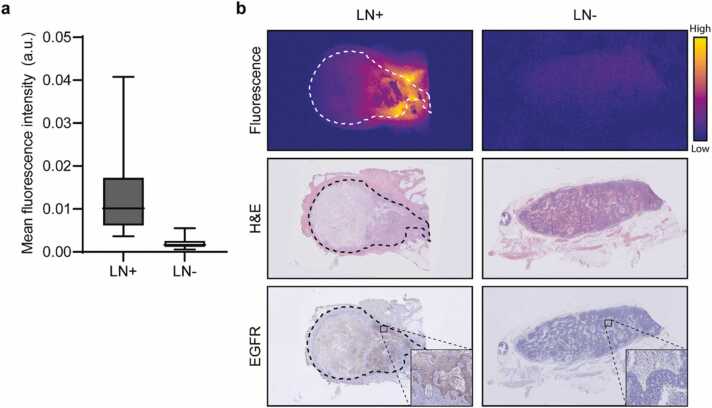
**Specimen analyses.** a) Malignant lymph nodes (LN)s show over six times increased mean fluorescence intensity compared to benign LNs (p < 0.0001). b) Representative macroscopic fluorescence images of a malignant and a benign LNs from a subject diagnosed with metastases upon final histopathology. The malignant LN shows increased fluorescence signal in the tumor tissue except in areas of necrosis. This cetuximab-800CW fluorescence largely co-localizes with EGFR-expression. Earlier, we showed the tumor-specificity of cetuximab-800CW fluorescence in LNs [48]. Abbreviations: LN+ , malignant lymph node; LN-, benign lymph node; H&E, hematoxylin and eosin; EGFR, epidermal growth factor receptor.

Analyzing the fluorescence images of all formalin-fixed LNs that were surgically excised during this study (n = 149), we observed significantly increased cetuximab-800CW concentrations in the malignant cases. Fig. 5a shows the increased mean fluorescence intensity of 1.02 (IQR 0.61–1.73) x 10^-2^ observed in malignant LNs (n = 9) compared to 0.16 (IQR 0.11–0.26) x 10^-2^ in benign LNs (n = 140) (p < 0.0001). Furthermore, malignant LNs showed a maximum fluorescence intensity of 2.49 (IQR 1.72–3.89) x 10^-2^ compared to 0.50 (IQR 0.37–0.74) x 10^-2^ in benign LNs (p < 0.0001), as shown earlier [48]. Fig. 5b shows a fluorescence image of a malignant LN that was localized against the submandibular gland, which allowed for correlation of MSOT with fluorescence molecular imaging and histology. A benign LN is shown as a reference.

## 4 Discussion

This study demonstrated MSOT for *in vivo* imaging of LNs in oral cancer patients. We characterized the optoacoustic properties of cetuximab-800CW using a tissue-mimicking phantom. Furthermore, we provided the first clinical results of *in vivo* EGFR-targeted molecular imaging with MSOT. Although we validated the suitability of cetuximab-800CW as a tumor-specific contrast agent for MSOT through our phantom and *ex vivo* experiments, we identified a number of obstacles preventing its reliable detection *in vivo*. Next, we compiled a list of recommendations for future studies. As a secondary goal, we assessed the qualitative and quantitative optoacoustic features of LNs in oral cancer patients. MSOT was able to detect altered tissue metabolism in malignant LNs, showcased by an increase in deoxyhemoglobin variance.

Our phantom experiment demonstrated the ability of MSOT to detect the EGFR-targeted fluorescent probe, cetuximab-800CW, at concentrations above 400 μM. Furthermore, the analysis of the excised specimens verified the tumor-specific intake of the tracer in LNs, confirming observations from previous fluorescence molecular imaging studies [35], [36], [48]. Recently, Nishio et al. [37] reported that a IRDye800CW-labeled antibody could be used for *ex vivo* optoacoustic detection of LN metastases. We could not extend these results by observing the accumulation of cetuximab-800CW in MSOT scans of LNs *in vivo*. We observed an increase in cetuximab-800CW signal only in one out of three examined cases, but here also big discrepancy between the pre- and post-injection imaging angles was observed. On the contrary, the case with the best alignment between the pre- and post-injection images did not show any change. Multiple reasons could explain the differences between our results and the previously reported *ex vivo* study. First, the light scattering and absorption in the overlying tissue present during *in vivo* imaging cause spectral coloring and signal decay due to reduced light fluence, complicating the detection of optical contrast in the LNs compared to *ex vivo* imaging. Secondly, the maximum permissible light fluence exposure for in-human use [40] is well below the laser power used by Nishio et al., further limiting the MSOT signal strength *in vivo*. Overall, we surmise that the selected dose of cetuximab-800CW lies below the detection limit of MSOT *in vivo* at depth, under the selected image acquisition parameters.

A secondary aim of this study was to explore MSOT of intrinsic contrast, particularly HbO_2_ and HbR. The *in vivo* imaging of HbR and HbO_2_ allowed the visualization of multiple endogenous features within and around LNs, such as lymphoid vasculature which seemed more extensively present in malignant LNs [51], [52]. Furthermore, we observed a significant increase in HbR variance in malignant LNs. The increased HbR heterogeneity in malignant LNs may be explained by the fact that malignant LNs contain both healthy tissue, that typically exhibits lower oxygen metabolism, and tumor tissue that is characterized by regions of hypoxia due to decreased oxygen supply (from dysfunctional microvasculature) and increased oxygen demand (from the hypermetabolic state of tumor cells) [31], [53], [54]. The non-invasive detection of malignant LNs with MSOT can help to better stratify oral cancer patients for surgical treatment of the neck and avoid overtreatment resulting from the limited sensitivity of current imaging methods. Moreover, the use of intrinsic contrast disposes the need for exogenous contrast agents, allowing for seamless implementation into standard of care.

This first clinical proof-of-concept study revealed several limitations of the chosen methodology. First, the difficulties in linking *in vivo* imaging to the final histopathology aggravated the already quite low sample size, prohibiting significant results. Unlike in studies assessing the performance of established techniques (*e.g.*, ultrasound), the novelty of MSOT LN imaging and the explorative nature of this study require meticulous correlation with histopathology before certain image characteristics can be attributed to the presence of tumor. Second, the mismatch of imaging positions between the pre- and post-injection scans complicates the comparison of chromophore quantities. Third, in this study we acquired images at six wavelengths matching the absorption spectrum characteristics of IRDye800CW. There are multiple other chromophores in the tissue and the spectral coloring increases the range of observable spectra even further, in which case six wavelengths do not facilitate reliable spectral unmixing. Finally, besides the challenges in the study procedure itself, the issue of detecting the contrast agent *in vivo* remains. We studied IRDye800CW as we could determine its potential noninvasively by including patients already administered with cetuximab-800CW as part of a fluorescence-guided surgery trial. However, better contrast agents could be considered to improve the tumor-specific signal generation for MSOT, such as gold nanoparticles [55]. The main criteria for optoacoustic contrast agents include strong and sharply peaked optical absorption in the near-infrared window, high optoacoustic efficiency, and optimal biocompatibility [56], [57]. Also, when evaluating novel optoacoustic contrast agents, it is helpful to realize that the accumulation of contrast agents involves complex pharmacokinetic processes, including both active binding of the target of interest (*e.g.*, receptor) as well as nonspecific accumulation due to variety in lymph and blood physiological processes [58], [59]. To better quantify target expression one could use a paired-imaging approach, where a non-targeted tracer with a different absorption spectrum is administered simultaneously and used to correct for nonspecific accumulation [60], [61].

Based on the limitations that we identified during our study, we compiled the following recommendations for future studies: 1) ensure node-by-node comparison by establishing specific study designs [62] or including only patients of which the LNs identified with preoperative imaging can tracked until final histopathology (*e.g.* preoperative lymphoscintigraphy and intraoperative Geiger meter-detection in the sentinel node procedure) [63], [64]; 2) when multiple sentinel LNs are identified, the endpoints should comprise the number of LNs identified, the metastatic state of the sentinel LN specimen and the number of malignant LNs; 3) a baseline MSOT could be performed to study pre- and post-injection scans; if a good match between pre- and post-injection scans cannot be established, multiple angles should be scanned and their results averaged; 4) additional wavelengths should be included in the image acquisition to increase the accuracy of spectral unmixing (*i.e*., at 25 wavelengths, the image acquisition takes 1 s and motion is thus not a problem, but the redundancy in the spectral dimension makes spectral unmixing more robust to noise and light fluence effects); 5) blind spectral unmixing (*e.g.*, non-negative matrix factorization) may be preferred to simple linear spectral unmixing when recording sufficient number wavelengths, as it can better adapt to observed spectral variations; 6) MSOT and, if the quantum yield of the of the contrast agent allows, fluorescence imaging of the excised and grossed LNs can be performed to validate *in vivo* imaging results. Here, MSOT of the excised LNs would require scanning in a water bath to improve acoustic coupling; 7) all sentinel lymph nodes should be examined through histopathology to determine their metastatic status. Finally, when suspicious features are determined based on MSOT, subsequent studies can correlate the number of identified LNs with final histopathology as in studies with ultrasound.

In conclusion, we identified possible reasons which prevent achieving the primary goal of detecting cetuximab-800 CW *in vivo*. Furthermore, we demonstrated that *in vivo* MSOT can observe clinically important features in LNs, specifically using hemoglobin parameters. In particular, increased variance of the deoxyhemoglobin distribution inside the LNs could serve as an optoacoustic marker of LN metastases. Additionally, we suggested several approaches to improve the efficiency of follow-up MSOT studies on LN imaging, such as a confined study population that allows correlation with histopathology, using more wavelengths during the image acquisition and the use of advanced spectral unmixing algorithms. Following our suggestions, future studies may better evaluate the clinical benefit of optoacoustic contrast agents as well as the intrinsic LN features observed with MSOT, facilitating improved preoperative detection of LN metastases.

## Declaration of Competing Interest

The authors declare the following financial interests/personal relationships which may be considered as potential competing interests, . JK, MB, DJ, and VN received funding from the European Research Council (ERC) under the European Union’s Horizon 2020 research and innovation programme under grant agreement No 694968 (PREMSOT) and by the 10.13039/501100001659Deutsche Forschungsgemeinschaft (DFG), Sonderforschungsbereich-824 (SFB-824), subproject A1. VN is an equity owner and consultant at iThera Medical GmbH, member of the Scientific Advisory Board at SurgVision BV / Bracco Sp.A, owner at Spear UG, founder and consultant at I3, and founder of Sthesis. GMvD is CEO, founder and shareholder of TRACER Europe BV / AxelaRx.
