# Supplementary material for: Multispectral optoacoustic tomography for in vivo detection of lymph node metastases in oral cancer patients using an EGFR-targeted contrast agent and intrinsic tissue contrast: A proof-of-concept study
Source: Photoacoustics. 2022 Apr 29;26:100362. doi: 10.1016/j.pacs.2022.100362 (PMC9079001; doi:10.1016/j.pacs.2022.100362)
Supplement: Supplementary file 1 — Supplementary material [file mmc1.docx]

# Supplemental materials

## Exclusion criteria

The exclusion criteria of the ICON study (NCT03134846) were concurrent uncontrolled medical conditions, inadequately controlled hypertension, receiving an investigational drug <30 days prior to scheduled tracer administration, an event of myocardial infarction, cerebrovascular accident, uncontrolled cardiac heart failure, significant liver disease or unstable angina <6 months prior to enrolment. Other exclusion criteria were a life expectancy <26 weeks, Karnofsky performance status <70%, pregnancy, history of infusion reactions to cetuximab or other monoclonal antibody therapies, QTc prolongation (i.e. >440 in males and >450 in females) and magnesium, potassium or calcium deviations of CTCAE grade II and higher.

## Supplementary figures


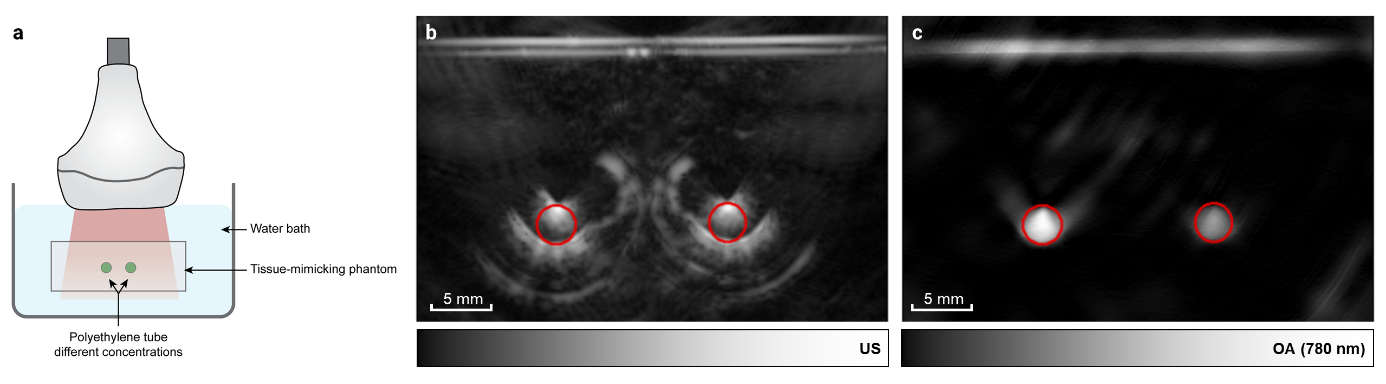


**Supplementary figure S1 | Phantom experiment.** a) Schematic overview of the phantom experiment. b) Ultrasound image of the phantom with two embedded tubes (marked by red circles). Acoustic impedance difference of the tubes and the surrounding agar causes reverberations producing visible artifacts. c) Optoacoustic image (λ=780 nm) of the two tubes (marked by red circles) with highest concentrations of cetuximab-CW800. Abbreviations: US, ultrasound; OA, optoacoustics.


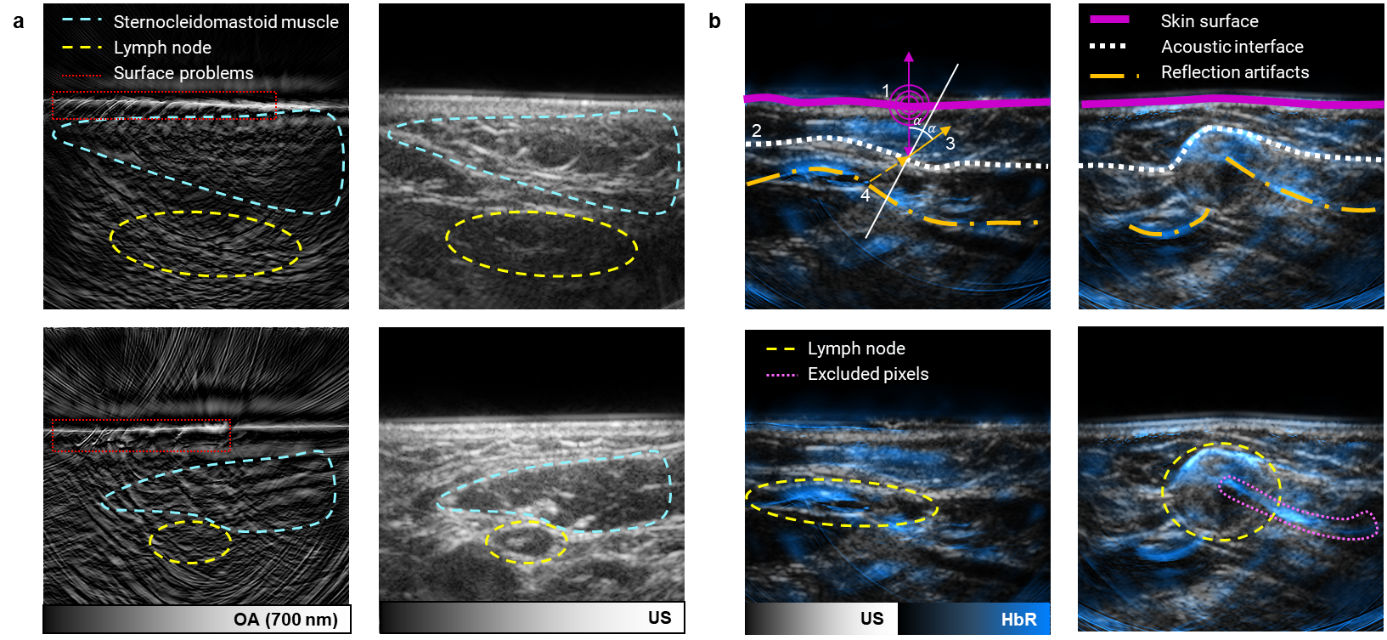


**Supplementary figure S2 | Image quality issues and reflection artifacts.** a) Two examples of surface contact issues (red dotted line) and occlusion of the lymph nodes (yellow dashed line) by the sternocleidomastoid muscle (aqua dashed line). Both problems contribute to bad image quality and too weak signal in the lymph node region, as seen in the left optoacoustic images ($\lambda$=700 nm). Ultrasound images are shown on the right. b) Two examples of the reflection artifacts and a scheme of the physical mechanism behind the phenomenon. An acoustic pressure pulse is emitted from an optical absorber in all directions (1). Part of the wave travels directly to the detectors, but part propagates into the tissue. Acoustic interfaces inside the tissue (2) act as reflectors. The wave gets reflected from the interface and travels outwards to the transducers (3). The reconstruction algorithm is oblivious to the reflections affecting the incoming waves and assumes the signal came along a straight line from an absorber at a distance given by the travel time and the speed of sound (4). A reflection artifact is thus observed behind the reflecting interface at a distance proportional to the distance between the interface and the true pressure wave origin. The most observed reflection is of the skin where the light fluence is highest, and thus, the optoacoustic signal is strongest. The skin reflection mainly affects the images of deoxyhemoglobin. This happens because the main absorber in the skin is melanin, whose absorption spectrum coincides with deoxyhemoglobin in the MSOT wavelength range. Abbreviations: LN+, malignant lymph node; LN-, benign lymph node.


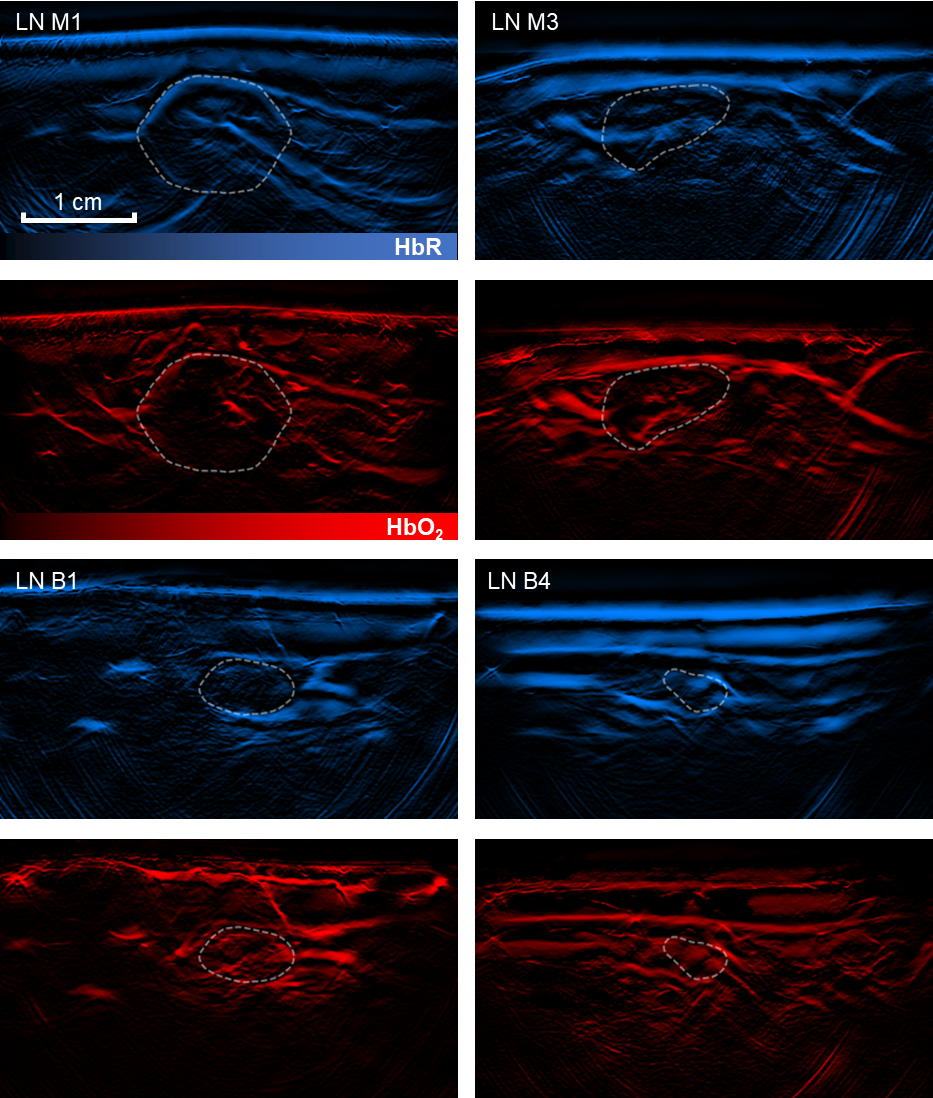


**Supplementary figure S3 | Intrinsic chromophore maps in malignant and benign lymph nodes.** Standalone visualizations of deoxyhemoglobin (HbR) and oxyhemoglobin (HbO_2_) maps in malignant lymph nodes M1 and M3 and benign lymph nodes B1 and B4.
